# Post-COVID interstitial lung disease in symptomatic patients after COVID-19 disease

**DOI:** 10.1007/s10787-023-01191-3

**Published:** 2023-03-24

**Authors:** Dorottya Fesu, Lorinc Polivka, Eniko Barczi, Marcell Foldesi, Gabor Horvath, Edit Hidvegi, Aniko Bohacs, Veronika Muller

**Affiliations:** 1 Department of Pulmonology, Semmelweis University, Tömő u. 25–29, 1083 Budapest, Hungary; 2 Neumann Medical Ltd, Buday László u. 12, 1024 Budapest, Hungary

**Keywords:** Post-COVID-19, Interstitial lung disease, COVID-19 pneumonia, Respiratory symptoms, High resolution CT, Lung function

## Abstract

COVID-19 is often associated with long-lasting pulmonary symptoms. Data are scarce about interstitial lung disease (ILD) in patients following COVID-19 hospitalization with persistent symptoms. We retrospectively reviewed all cases sent to pulmonary post-COVID evaluation due to persistent symptoms between February 2021 and February 2022 (N = 318). All patients with suspected ILD (N = 44) were reviewed at the multidisciplinary discussion. Patient characteristics, symptoms, time since hospitalization, detailed lung function measurements and 6-min walk test (6MWT) were evaluated. The post-COVID ILD suspected group included more men (68.2 vs. 31.8%) with significantly older age compared to the control group (64.0 ± 12.3 vs. 51.3 ± 14.9 years). Most patient needed hospital care for COVID-19 pneumonia (68.6% of all patients and 84.1% of ILD suspected group) and average time since hospitalization was 2.4 ± 2.3 months. Persisting symptoms included fatigue (34%), dyspnoea (25.2%), cough (22.6%), and sleep disorders (insomnia 13.2%; sleepiness 8.2%). Post-COVID ILD presented more often with new symptoms of cough and sleepiness. Functional impairment, especially decreased walking distance and desaturation during 6-min walk test (6MWT) were detected in the ILD-suspected group. Respiratory function test in the post-COVID ILD group showed slight restrictive ventilatory pattern (FVC: 76.7 ± 18.1%, FEV1: 83.5 ± 19.1%, TLC: 85.6 ± 28.1%) and desaturation during 6MWT were detected in 41% of patients. LDCT changes were mainly ground glass opacities (GGO) and/or reticular abnormalities in most cases affecting < 10% of the lungs. Our data indicate that suspected post-COVID ILD is affecting 13.8% of symptomatic patients. High resolution chest CT changes were mainly low extent GGO/reticulation, while long-term lung structural changes need further evaluation.

## Introduction

Interstitial lung diseases (ILD-s) are often progressive and result in structural alterations. Lung fibrosis and decreased functional lung capacity severely affect patients’ life-quality and outcome (Raghu et al. 2022).

Lung infections, especially viruses and intracellular pathogens alter alveolar epithelial cells, and the regeneration of the alveoli might be delayed or altered. Aberrant healing processes might also result in ILD-s which might progress into fibrosis. (Atabati et al. 2020; McDonald 2021; Naik and Moore 2010) Patients infected by severe acute respiratory syndrome-related coronavirus 2 (SARS-CoV2) might present with reactive epithelial changes and diffuse alveolar damage (DAD); and acute organizing pneumonia which may heal without residual damage, or in still not well defined proportion lead to fibrotic alteration, or acceleration of fibrosis in already known or unknown underlying ILD (John et al. 2021).

Long-COVID or post-COVID syndrome is defined as a long-term condition, with several manifestations of a multisystem disease including different persisting symptoms, following mild-severe coronavirus disease 19 (COVID-19) (CDC 2021). The most common symptoms studies have mentioned are mainly pulmonary, including dyspnoea, cough, chest pain, decreased exercise capacity, fatigue and sleep disturbances (Mandal et al. 2021; Huang et al. 2021).

Radiological changes of COVID-19 pneumonia might persist over longer term, and the resolution of alveolar and interstitial alterations of infection is not uniform. Persisting radiological abnormalities include ground-glass opacity (GGO) and fibrotic-like (reticulation) changes, and these were more common in severe cases and were associated with mechanical ventilation according to previous studies (McGroder et al. 2021; Lerum et al. 2021).

Long term consequences of COVID pneumonia are still not known, however long-lasting interstitial lung abnormalities including GGO, and reticulation need further investigations. Our aim was to assess frequency of ILD among long-COVID patients; and to analyse symptom profile and functional status of post-COVID ILD and control patients referred to our post-COVID pulmonary care.

## Methods

### Study population

Our study is based on a retrospective data evaluation and analysis of 318 outpatients who were admitted to the post-COVID-19 pulmonary care-system between February 2021 and February 2022 at the Department of Pulmonology, Semmelweis University. The long-COVID-19 pulmonary care was established to follow and investigate persisting complaints and symptoms of patients; and to give a proper and adequate support after confirmed COVID-19 infection. Patients with persisting symptoms could enter the post-COVID-19 care in the following cases: at least 4–8 weeks after their dismission from the Pulmonology Department’s COVID-19 wards; at least 8–12 weeks after their discharge from any other hospital’s COVID-19 wards; and at least 8–12 weeks after their infection with persisting symptoms following proven COVID-19 disease treated at home referred from outpatient pulmonary care.

Data collection, storage, syntactic and semantic validation were performed using a workflow-integrated structured data collection and analysis software provided by Neumann Medical Ltd.

Data on previous medical conditions; COVID-19 related history; symptoms during COVID and at time of the visit; physical examination; 6-min-walk test (6MWT); low-dose high resolution chest-CT (LDCT) and respiratory function tests were registered. Respiratory function tests included measurement of forced vital capacity (FVC); forced expiratory volume in 1 s (FEV1); FEV1/FVC and total lung capacity (TLC) according to the American Thoracic Society and European Respiratory Society (ATS/ERS) guidelines (Graham et al. 2017, 2019). The diffusing capacity for carbon monoxide (TLCO) was measured with the single breath CO method. Furthermore, the transfer coefficient for carbon monoxide (KLCO) was calculated (PDD-301/s, Piston, Budapest, Hungary). Indices of respiratory muscle strength Pi max and Pe max were registered.

Data about patients’ hospital stay during COVID-19 infection, including chest CT results, oxygen supplementary need, antiviral and COVID specific therapy (favipiravir, remdesivir, covalescent plasma, steroids, anticoagulants and antibiotics), and need for non-invasive or invasive ventilation were collected. All patients with persisting changes on the LDCT (ILD-suspected group) were referred to a multidisciplinary team discussion (MDD) and the MDD was responsible for the diagnosis-process and made suggestions for further examinations and therapy.

The patient population was divided into two groups for statistical analysis (non-ILD vs. ILD-suspected). The two groups were compared for differences in anthropometric data, symptoms, respiratory tests, and additional therapy. The study was designed in accordance with the 1964 Helsinki declaration and its later amendments, and the protocol was approved by the ethical committee of the Semmelweis University Regional and Institutional Committee of Science and Research Ethics (SE RKEB 145/2022).

### Statistical analysis

Statistical analysis was performed using IBM SPSS statistical program and Microsoft Excel. Differences in categorical variables between groups were evaluated by Chi-squared test and two-tailed Fisher’s exact test. Continuous variables were described as the mean ± standard deviation, and Student’s t test was applied for comparing the continuous data between groups. The p value < 0.05 was defined as statistically significant.

## Results

Main results about patient characteristics; lung function and 6MWT results; symptoms and data about the used therapy are summarized in Table 1.

**Table 1:** Patient characteristics; lung function results; 6MWT results; symptoms; used therapy

Patient characteristics	ALL	ILD suspected	Non-ILD suspected	p values
N	318	44	274	–
Age	53.1 ± 15.2	64.0 ± 12.3	51.3 ± 14.9	** < 0.001**
Sex (female:male)	133:185	14:30	119:155	0.147
Time since hospital discharge (months)	2.6 ± 2.3	2.4 ± 2.3	2.7 ± 2.3	0.501
Outpatients	100 (31.4%)	7 (15.9%)	93 (33.9%)	**0.017**
BMI (kg/m^2^)	30.2 ± 7.1	29.1 ± 4.2	30.0 ± 6.8	0.168
No BMI data	21 (7.7%)	0 (0%)	21 (6.6%)	–
Lung function test				
FVC (L)	3.4 ± 1.0	3.0 ± 1.0	3.5 ± 1.0	**0.009**
FVC (%)	82.7 ± 16.3	76.7 ± 18.1	83.8 ± 15.7	**0.018**
FEV1 (L)	2.9 ± 0.9	2.5 ± 0.8	2.9 ± 0.9	**0.004**
FEV1 (%)	87.3 ± 17.7	83.5 ± 19.1	87.9 ± 17.4	0.171
FEV1/FVC	77.4 ± 23.3	66.7 ± 33.4	79.3 ± 20.5	**0.024**
TLC (L)	5.7 ± 4.0	6.5 ± 9.6	5.6 ± 1.6	0.528
TLC (%)	94.3 ± 24.6	85.6 ± 28.1	95.8 ± 23.7	**0.035**
RV (L)	2.15 ± 1.6	2.1 ± 0.9	2.2 ± 1.6	0.604
RV (%)	99.6 ± 49.1	82.2 ± 40.6	103.3 ± 50.0	**0.014**
FEF25% (L/sec)	6.2 ± 2.04	6.0 ± 1.9	6.3 ± 2.1	0.324
FEF25% (%)	92.9 ± 5.3	88.8 ± 24.2	93.8 ± 25.6	0.294
FEF50% (L/sec)	3.6 ± 1.3	3.2 ± 1.0	3.7 ± 1.3	**0.007**
FEF50% (%)	81.4 ± 28.9	75.1 ± 25.5	82.7 ± 29.5	0.136
FEF75% (L/sec)	1.2 ± 0.6	0.9 ± 0.4	1.2 ± 0.6	** < 0.001**
FEF75% (%)	120.4 ± 62.7	133.5 ± 75.7	118.1 ± 60.0	0.223
FEF25-75% (L/sec)	3.5 ± 2.6	2.9 ± 1.0	3.6 ± 2.8	**0.002**
FEF25-75% (%)	113.3 ± 37.1	118.7 ± 39.7	112.3 ± 36.7	0.347
Raw (kPa*s/L)	0.4 ± 2.7	0.2 ± 0.1	0.5 ± 2.9	0.231
TLCO (mmol/min/kPa)	9.3 ± 5.3	7.2 ± 2.3	9.7 ± 5.6	** < 0.001**
TLCO (%)	103.8 ± 26.9	85.0 ± 21.2	107.1 ± 26.5	** < 0.001**
KLCO (mmol/min/kPa/L)	2.1 ± 8.1	4.6 ± 20.7	1.7 ± 0.4	0.375
KLCO (%)	105.5 ± 26.8	89.5 ± 24.7	108.4 ± 26.2	** < 0.001**
Pimax (kPa)	7.8 ± 3.4	7.9 ± 3.5	7.8 ± 3.4	0.914
Pemax (kPa)	9.2 ± 3.4	9.1 ± 3.8	9.2 ± 3.3	0.794
6MWT results				
6 MW distance (m)	455.1 ± 113.1	395.6 ± 159.6	464.9 ± 100.5	**0.0110**
Heart rate at start (1/min)	85.67 ± 13.9	85.8 ± 13.3	85.7 ± 14.0	0.9660
Heart rate at end (1/min)	199.1 ± 22.4	118.5 ± 24.3	119.2 ± 22.1	0.8490
Saturation at start (%)	96.3 ± 1.9	95.9 ± 1.5	96.4 ± 2.1	0.3400
Saturation at end (%)	89.5 ± 6.7	88.0 ± 7.9	90.1 ± 6.2	0.3200
Patients with desaturation n (%)	67 (21.1%)	18 (40.9%)	49 (17.9%)	**0.0005**
BORG at start (0–10) (median)	0.0 ± 7.8	0.0 ± 5.3	0.0 ± 8.1	0.0640
BORG at end (0–10) (median)	10.0 ± 15.2	5.0 ± 14.0	10.0 ± 15.4	0.5040
Symptoms				
Patients with discontinued symptoms since hospital discharge: n (%)				
Fever, chills	210 (66.0%)	24 (54.5%)	186 (67.9%)	0.083
Cough	137 (43.1%)	16 (36.4%)	121 (44.2%)	0.332
Dyspnoea	133 (41.8%)	23 (52.3%)	110 (40.1%)	0.130
Fatigue	125 (39.3%)	15 (34.1%)	110 (40.1%)	0.445
Muscle pain	115 (36.2%)	7 (15.9%)	108 (39.4%)	**0.003**
Sleepiness	97 (30.5%)	10 (22.7%)	87 (31.8%)	0.227
Insomnia	62 (19.5%)	8 (18.2%)	54 (19.7%)	0.813
Headache	100 (31.4%)	8 (18.2%)	92 (33.6%)	**0.041**
Palpitation	79 (24.8%)	7 (15.9%)	72 (26.3%)	0.140
Loss of smell or taste	89 (28.0%)	13 (29.5%)	76 (27.7%)	0.804
Sore throat	88 (27.7%)	7 (15.9%)	81 (29.6%)	0.060
Runny nose	89 (28.0%)	8 (18.2%)	81 (29.6%)	0.119
Nausea	53 (16.7%)	5 (11.4%)	48 (17.5%)	0.309
Vomiting	50 (15.7%)	6 (13.6%)	44 (16.1%)	0.682
Diarrhoea	90 (28.3%)	11 (25.0%)	79 (28.8%)	0.600
Patients with persisting symptoms since hospital discharge: n (%)				
Fever, chills	10 (3.1%)	1 (2.3%)	9 (3.3%)	0.721
Cough	72 (22.6%)	9 (20.5%)	63 (23.0%)	0.709
Dyspnoea	80 (25.2%)	9 (20.5%)	71 (25.9%)	0.439
Fatigue	108 (34%)	13 (29.5%)	95 (34.7%)	0.505
Muscle pain	36 (11.3%)	5 (11.4%)	31 (11.3%)	0.992
Sleepiness	26 (8.2%)	2 (4.5%)	24 (8.8%)	0.344
Insomnia	42 (13.2%)	7 (15.9%)	35 (12.8%)	0.569
Headache	18 (5.7%)	2 (4.5%)	16 (5.8%)	0.730
Palpitation	42 (13.2%)	8 (18.2%)	34 (12.4%)	0.294
Loss of smell or taste	26 (8.2%)	2 (4.5%)	24 (8.8%)	0.344
Sore throat	5 (1.6%)	0 (0%)	5 (1.8%)	0.366
Runny nose	29 (9.1%)	4 (9.1%)	25 (9.1%)	0.994
Nausea	3 (0.9%)	2 (4.5%)	1 (0.4%)	**0.008**
Vomiting	2 (0.6%)	1 (2.3%)	1 (0.4%)	0.137
Diarrhoea	8 (2.5%)	2 (4.5%)	6 (2.2%)	0.354
Patients with new symptoms since hospital discharge: n (%)				
Fever, chills	1 (0.3%)	0 (0%)	1 (0.4%)	0.688
Cough	11 (3.5%)	5 (11.4%)	6 (2.2%)	**0.002**
Dyspnoea	12 (3.8%)	2 (4.5%)	10 (3.6%)	0.772
Fatigue	12 (3.8%)	2 (4.5%)	10 (3.6%)	0.772
Muscle pain	11 (3.5%)	3 (6.8%)	8 (2.9%)	0.189
Sleepiness	11 (3.5%)	4 (9.1%)	7 (2.6%)	**0.028**
Insomnia	17 (5.3%)	2 (4.5%)	15 (5.5%)	0.800
Headache	7 (2.2%)	2 (4.5%)	5 (1.8%)	0.254
Palpitation	14 (4.4%)	2 (4.5%)	12 (4.4%)	0.960
Loss of smell or taste	1 (0.3%)	0 (0%)	1 (0.4%)	0.689
Sore throat	6 (1.9%)	1 (2.3%)	5 (1.8%)	0.839
Runny nose	9 (2.8%)	2 (4.5%)	7 (2.6%)	0.460
Nausea	5 (1.6%)	1 (2.3%)	4 (1.5%)	0.162
Vomiting	4 (1.3%)	1 (2.3%)	3 (1.1%)	0.515
Diarrhoea	3 (0.9%)	1 (2.3%)	2 (0.7%)	0.326
COVID-19 therapy: n (%)				
Antibiotics	219 (69.9%)	35 (79.5%)	184 (67.2%)	0.099
Favipiravir	46 (14.5%)	8 (18.2%)	38 (13.9%)	0.450
Remdesivir	145 (45.6%)	25 (56.8%)	120 (43.8%)	0.107
Covalescent plasma	17 (5.3%)	3 (6.8%)	14 (5.1%)	0.640
Steroids	221 (69.5%)	37 (84.1%)	184 (67.2%)	**0.024**
Anticoagulants	218 (68.6%)	36 (81.8%)	182 (66.4%)	**0.041**
Oxygen	208 (65.4%)	36 (81.8%)	172 (62.8%)	**0.014**
Non-invasive ventilation support	57 (17.9%)	9 (20.5%)	48 (17.5%)	0.637
Invasive ventilation support	15 (4.7%)	1 (2.3%)	14 (5.1%)	0.410

Significant difference between ILD-suspected and Non-ILD suspected groups are highlighted in bold. The p value < 0.05 was defined as statistically significant

The patient population with suspected ILD was older and needed hospitalization during their COVID-19 disease more often than the control group. There were no significant differences in sex, however both, the post-COVID control and post-COVID ILD suspect groups, included more men. Most patients were overweight.

In the control group, significantly more patients reported that the following symptoms disappeared after illness: muscle aches (15.9% vs. 39.4%); headache (18.2% vs. 33.6%). Regarding the persisting symptoms (which persisted at the time of patient’s presentation at post-COVID pulmonary care), the most common were fatigue (34.0%), dyspnoea (25.2%) and cough (22.6%), with no significant difference between the ILD suspect and control groups. These symptoms were followed by sleep disturbances (insomnia 13.2%; sleepiness 8.25%). In terms of new onset symptoms, insomnia (5.3%); palpitation (4.4%), fatigue (3.8%) and dyspnoea (3.8%) were the most frequently reported. Cough (11.4% vs. 2.2%); and sleepiness (9.1% vs. 2.6%) were significantly more frequent new symptoms in patients with suspected ILD compared to the control group.

Among patients with suspected ILD, significantly reduced FVC; FEV1; FEV1/FVC; TLC; RV, TLCO and KLCO parameters were measured. Patients with suspected ILD were able to walk a significantly shorter distance and showed desaturation significantly more often (40.9% vs. 17.9%) during the 6MWT.

In our study the most common LDCT findings were GGO and reticulation involving less than 10% of the lung parenchyma.

## Discussion

Our study confirmed that high proportion of patients have persisting or new pulmonary symptoms following COVID-19 infection. HRCT changes were present in 13.8% of patients, who need follow-up of lung abnormalities.

The proportion of patients with ILD was lower than in a UK study (Myall et al. 2021), in which 24% of patients were suspected of having ILD. In that study, CT abnormalities were identified in 76.6% of patients referred for MDD, with the highest rates of organizing pneumonia and GGO. Based on the MDD diagnosis, ILD was diagnosed in 4.8% of the total patient population (837 patients). Patients with ILD were started on steroid therapy, and a subsequent follow-up study assessed the reduction of previous symptoms and described the regression of CT abnormalities; highlighting that CT lesions did not progress to fibrosis in patients treated with steroids (Myall et al. 2021). In our patients steroids were used during the hospital stay in high proportion as standard of care treatment, non-treated patients were mainly from the group of referrals who had their infection at home (Polivka et al. 2022). This might have contributed to the lower number of pulmonary changes on LDCT.

In our study, the most frequently observed symptom was fatigue, which persisted in one third of patients. Fatigue was also the leading symptom in all previous studies. The second most common symptom, affecting about a quarter of patients, was respiratory in origin. The most common were dyspnoea (25.2% as a persistent symptom); and cough (22.6% as a persistent symptom), and the third most common complaint was sleep disturbance (insomnia 13.2% and sleepiness 8.2% as a persistent symptom). ILD suspected group reported more often cough and sleep disorders.

Several other studies described the prevalence of post-COVID symptoms. A Chinese study described fatigue in 63% of patients 6 months after COVID-19; sleep disturbance in 26%; and anxiety and/or depression in 23% (Huang et al. 2021). Also in an Austrian study, 41% of patients experienced symptoms after the infection had passed; the most common symptoms being dyspnoea (36%); night sweats (24%) and sleep disturbance (22%). In this study, at a later follow-up visit (100 days after the onset of infection), patients had a reduced rate of symptoms, indicating that post-COVID syndrome is a dynamically changing and generally improving condition (Sonnweber et al. 2021).

Our study also compares the symptoms of patients with suspected ILD with those of controls (non-ILD patients) who have undergone COVID-19. The difference between the two groups was significant for new-onset symptoms, with cough and sleepiness being more frequent in patients with suspected ILD. This may suggest that the persistence of these two symptoms and a history of COVID-19 disease may raise the clinicians’ attention for the possibility of lung abnormalities, including ILD.

Lesions with characteristics of ILD after COVID-19 disease can be identified in varying degrees and patterns in affected individuals. The most typical patterns are GGO, reticulation and consolidation (Sonnweber et al. 2021; Lerum et al. 2021; McGroder et al. 2021; Besutti et al. 2022; Liu et al. 2021) which are rarely associated with fibrosis immediately after the infection and often show regression (Mandal et al. 2021; Sonnweber et al. 2021; Myall et al. 2021). Two international studies highlight organizing pneumonia (Myall et al. 2021) and usual interstitial pneumonia (UIP) (Konopka et al. 2021) as the most common patterns. Many cases with CT lesions are not associated with respiratory symptoms or impairment of lung function or 6MWT; therefore, Sonnweber et al. propose the use of CT scans for diagnosis or evaluation of post-COVID ILD instead of functional measurements (Sonnweber et al. 2021). Abnormal patterns in the lung parenchyma have been associated with the length of invasive ventilation, which may damage lung tissue due to a possible barotrauma; or could also be associated with the more severe form of COVID-19 in patients receiving invasive ventilatory support, which has been correlated with CT abnormalities in several studies (Huang et al. 2021)(Guler et al. 2021).

Regarding respiratory function results, the currently available literature is consistent in describing a pattern of restrictive ventilatory dysfunction (reduced TLC, FVC, RV) and diffusion reduction (TLCO; KLCO) (Huang et al. 2021; Lerum et al. 2021; Sonnweber et al. 2021; Guler et al. 2021; Myall et al. 2021; Stockley et al. 2021). Several studies have highlighted the impairment in diffusion parameters, which may be related to the severity of COVID-19 disease (Huang et al. 2021; Guler et al. 2021), the presence of CT lesions (McGroder et al. 2021), and the use of mechanical ventilation (Guler et al. 2021). In the present study, impairment in respiratory function and decreased diffusion capacity were also measured in the ILD suspect group, with a significant difference compared to the control group, where the decrease in these parameters was not significant. Regarding performance, 6MWT parameters, including desaturation and reduced distance parameters are characteristics of patients with suspected ILD, similar to previous observations (McGroder et al. 2021; Guler et al. 2021).

The exact prevalence of post-COVID disease is not yet known, and many studies have aimed to identify risk factors and predisposing factors. The international literature associates female sex (Huang et al. 2021), older age (Huang et al. 2021), obesity (Stockley et al. 2021), invasive ventilatory support (Lerum et al. 2021) and more severe COVID-19 disease (Huang et al. 2021) with the disease, while steroid therapy is considered as a beneficial factor (Myall et al. 2021). In contrast, in the present study, male sex is more prevalent in both groups, with no significant difference between the two groups. The ILD suspected patient group did not have a higher prevalence of invasive ventilatory support; and there was no difference in obesity as a risk factor compared to the control group. Regarding age, in accordance with the literature, the mean age was higher in the ILD suspect group, which might have contributed to the observed differences. Steroid therapy, oxygen supplementation need, and anticoagulant therapy were more frequent in the ILD suspect group, which can be associated with the fact that this patient group was more frequently admitted to hospital for COVID-19 infection, where these therapies are widely used according to guidelines.

Limitation of our study is the short follow-up for a limited number of patients and the lack of re-evaluation of LDCT scans and function.

## Conclusions

In our pulmonary care 13.8% of post-COVID patients had ILD based on LDCT.

Post-COVID ILD patients were older; presented more often with new symptoms of cough and sleepiness.

Functional impairment, especially decreased diffusion capacity and desaturation during 6MWT are important factors when ILD is suspected. 6MWT and lung function tests should be performed also at follow-up visits.

Post-COVID ILD patients were more often hospitalized, needed more intensive COVID-19 treatment including oxygen supplementation, steroids, and anticoagulants.

To assess long-term lung functional or radiological changes follow-up of ILD-suspected patients is planned.

## Data Availability

The datasets generated during and/or analysed during the current study are not publicly available due to continuing follow-ups but are available from the corresponding author on reasonable request.
